# Comparison of virulence factors between invasive and non-invasive clinical isolates of *Candida* spp.

**DOI:** 10.22034/cmm.2025.345248.1628

**Published:** 2025-08-26

**Authors:** Bryan Zamora, Lizeth Salazar, Marcelo Grijalva, Tatiana Lara, Patricia Jiménez, María José Vallejo-López

**Affiliations:** 1 Research Group on Animal and Human Health, Human Biotechnology Laboratory, Department of Life and Agricultural Sciences, Universidad de las Fuerzas Armadas ESPE, Sangolquí, Ecuador; 2 Center for Nanoscience and Nanotechnology, Department of Life and Agricultural Sciences, Universidad de las Fuerzas Armadas ESPE, Sangolquí, Ecuador; 3 Faculty of Medicine, University of the Americas, Quito, Ecuador

**Keywords:** *Candida*, Disseminated mycoses, Virulence factors

## Abstract

**Background and Purpose:**

Invasive fungal infections have high morbidity and mortality rates, with *Candida* species being the leading cause in hospitalized patients. Virulence factors, such as adhesion, enzyme secretion, and biofilm formation, play a major role in *Candida* pathogenesis. This study hypothesized that virulence factors in localized Candida infections behave differently than those in systemic infections.

**Materials and Methods:**

This study compared invasive and non-invasive *Candida* clinical isolates in terms of biofilm formation and enzymatic activity in. Biofilm mass and metabolic activity were assessed using crystal violet and XTT assays, while phospholipase and protease activities were measured in specific media. Qualitative biofilm characterization was performed using scanning electron microscopy and scanning confocal laser microscopy.

**Results:**

*Candida* isolates from invasive infections showed higher bulk biofilm production and metabolic activity, compared to localized infection isolates. Bulk biofilm production and metabolic activity were notably higher in systemic infections, compared to those in localized infections. Non-*albicans* *Candida* species exhibited a higher biofilm-forming capacity and metabolic activity, emphasizing their potential for more invasive infections. Conversely, hydrolytic enzyme production was higher in localized infection isolates, compared to that in systemic infection. Phospholipase activity showed no significant differences.

**Conclusion:**

The differences in enzymatic activity observed in isolates from various anatomical sites underscores the importance of considering the infection context in assessing virulence These findings highlight the role of proteases and other factors in *Candida* invasiveness, contributing new insights into *Candida* virulence factors in Ecuador.

## Introduction

*Candida* species are opportunistic human pathogens and among the leading causes of fungal infections in hospitalized patients. *Candida* bloodstream infections rank as the fourth most common nosocomial bloodstream infections, particularly affecting critically ill intensive care unit (ICU) patients [ 1 , 2 ]. Host-related risk factors, such as advanced age, comorbidities, central venous catheter presence, prolonged antibiotic use, immunodeficiencies, organ transplantation, and chemotherapy, contribute significantly to susceptibility [ 3 ]. Most candidemia and invasive candidiasis cases are caused by *C. albicans*, *C. glabrata*, *C. tropicalis*, *C. parapsilosis*, and *C. krusei*, which collectively represent about 90% of bloodstream isolates. *Candida*, a natural component of human microbiota, can shift from a commensal to pathogenic form due to the expression of virulence factors, such as adherence, secretion of hydrolytic enzymes, and biofilm formation [ 4 ]. Production of hydrolytic enzymes, such as phospholipases, lipases, and proteases, plays a critical role in tissue colonization and invasion [ 5 , 6 ]. 

Incidence of invasive candidiasis has remained relatively constant in Western countries, with variation by geographic area and patient demographics [ 3 ]. There are few reports on population studies in developing countries (Africa, Asia, and Latin America), making it difficult to estimate the exact prevalence of *Candida* spp. infections in these regions [ 7 ]. Available data suggest an increased mortality rate in Latin America, compared to the United States and Europe [ 8 - 14 ].

In Ecuador, limited studies have addressed *Candida* species identification, antifungal susceptibility [ 15 , 16 ], infection prevention [ 17 ], or ICU microbiological surveillance [ 18 ]. This study aimed to characterize virulence factors (*i.e.*, enzymatic activity and biofilm formation) in invasive and non-invasive clinical *Candida* isolates.

## Materials and Methods

### Candida spp. strains and culture conditions

Between 2018 and 2020, a total of 136 *Candida* spp. isolates from systemic and localized mycoses were collected from two referral hospitals in Quito, Ecuador. The study protocol was approved by the respective institutional Ethics Committees. Initial identification utilized the Vitek Yeast Biochemical Card (bioMérieux, France) and Germ Tube Test, followed by confirmation using the Vitek 2 analyzer and CHROMAgar plates.

The isolates were stored at the Nanomedicine and Nanobiology Laboratory at the University of the Armed Forces (ESPE). Culturing involved yeast peptone dextrose (YPD) broth (Beckton Dickinson, Maryland, USA/Le Pont de Claix, France) incubation at 37 °C for 24 h at 100 rpm followed by subculture on YPD agar (Sigma-Aldrich, St. Louis, MO, USA) for 24 h at 37 °C and cryopreservation in 50% (vol/vol) glycerol at -80 °C. For virulence testing, cells were cultivated in YPD agar and broth as described.

### Biofilm formation

Biofilm formation was induced according to previous protocols [ 19 ]. First, 1 mL of cellular suspension was centrifuged at 5,000 rpm for 6 min. The supernatant was discarded, and the cells were resuspended in RPMI-1640 medium (Corning, Manassas, USA) without bicarbonate, supplemented with L-glutamine and buffered with 0.165 M 3-(N-morpholino)propanesulfonic acid at pH 7.0, to a concentration of 1 × 10^6^ cells/mL. Cell suspensions were then seeded in flat-bottom 96-well microtiter plates (Corning, Kennebunk, USA) and incubated for 48 h at 37 °C. 

### Crystal violet staining

Biofilms were allowed to develop for 48 h, and then a crystal violet assay was performed according to the methodology developed by Jin *et al*. [ 20 ] with minor modifications. Each well was washed twice with 200 µL of phosphate-buffered saline (PBS) and air-dried for 20 min at 35 °C. Next, wells were stained with 110 µL of 0.4% aqueous crystal violet solution (Corning, Kennebunk, USA) for 45 min, then washed four times with 300 µL of sterile distilled water and once with 200 µL of 95% ethanol (destaining solution). After 45 min, 100 µL of the destaining solution from each well was transferred to a new microtiter plate. Absorbance values were measured using a microplate reader (Multiskan® GO, Thermo Scientific, Waltham, USA) at 595 nm. The average OD_595_ of the wells without cells (blank) was subtracted from each sample's average OD_595_ to eliminate background, with four technical replicates performed for each isolate.

### XTT reduction assay

The biofilm metabolic activity was assessed using the XTT-menadione reduction assay (XTT, Sigma-Aldrich, Eugene, USA), as described by other authors [ 20 , 21 ] Each well was washed with 200 µL of PBS, followed by the addition of 200 µL of PBS and 12 µL of the XTT-menadione solution. The microtiter plate was incubated at 37°C for 3 hours, protected from light. After incubation, 100 µL of the solution was transferred to a new plate. Absorbance values were measured in a microplate reader (Multiskan® GO, Thermo Scientific, Waltham, USA) at 490 nm. Four technical replicates were performed for each isolate. 

### Determination of phospholipase production

Phospholipase activity was evaluated using the method developed by *Price et al*. [ 22 ] with modified Sabouraud-dextrose agar (Liofilchem, Roseto d'Abruzzi, Italy) containing 8% egg yolk emulsion (Sigma-Aldrich, St. Louis, USA), 1 M NaCl, and 5 mM CaCl_2_. The isolates were first cultivated in YPD broth for 18 h at 37 °C and 100 rpm with an initial density of 1 × 10^6^ cells/mL. Next, 1 µL of each isolate was transferred to the test medium and incubated for 72 h at 37 °C. To calculate *Pz* values, the diameter of the colony was divided by the total diameter (colony plus precipitation zone). The *Pz* values near zero indicate high enzymatic activity, while values near 1 indicate low or no activity [ 22 ]. Each isolate was tested in duplicate.

### Determination of proteinase production

Proteinase production was assessed using yeast carbon base agar (Sigma-Aldrich, St. Louis, USA) with 0.2% yeast carbon base–bovine serum albumin as described by Rüchel et al. [ 23 ]. The *Pz* values were calculated as described above, with each isolate tested in duplicate. 

### Scanning Electron Microscopy

Six isolates from systemic and localized infections with high biofilm metrics were selected. Biofilms were grown on sterile coverslips and visualized using a Tescan Mira 3 scanning electron microscopy (SEM) at 10 kV, following Melo et al. [ 21 ] with minor modifications.

### Confocal Laser Scanning Microscopy

One *C. albicans* isolate per infection type was selected for biofilm structure analysis. The biofilms were developed in 35-mm-diameter glass-bottom dishes (Greiner Bio-One, Frickenhausen, Germany) for 48 h at 37 °C as described above. First, consecutive washes with PBS (Corning, Manassas, USA) were performed to remove planktonic cells. Afterward, 1 mL of Concanavalin A-Alexa Fluor 488 conjugate (Invitrogen, Eugene, USA) at a concentration of 10 µg/mL was added and incubated at 37 °C for 30 min. After staining, samples were visualized using a confocal laser scanning microscope (Olympus Fluoview FV1200/IX83, Hamburg, Germany) with a UPLSAPO 60x/N.A. 1.35 oil immersion objective lens, equipped with a multiline argon laser tuned to 488 nm. Biofilm thickness was quantified with COMSTAT software [ 24 , 25 ].

### Statistical analysis

Statistical significance was analyzed using an unpaired Student t-test with InfoStat software. Statistical significance thresholds were set at *p* < 0.05, 0.01, 0.001, and 0.0001.

## Results

### Distribution of Candida species

Among 136 isolates, *C. albicans* was the most common species (63.97%, n = 87), followed by *C. tropicalis* and *C. glabrata* (8.82% each), *C. parapsilosis* (8.09%), *C. ciferrii* and *C. lusitaniae* (3.68% each), and *C. krusei* and *C. guilliermondii* (1.47% each) (Table 1).

**Table 1:** Distribution of *Candida* species by infection type

Specie	Infection type		Total
	Localized infection	Systemic infection	
*Candida albicans*	54 (79.41%)	33 (48.53%)	87 (63.97%)
***Non-albicans Candida***			
*Candida tropicalis*	2 (2.94%)	10 (14.71%)	12 (8.82%)
*Candida glabrata*	5 (7.35%)	7 (10.29%)	12 (8.82%)
*Candida krusei*	0 (0%)	2 (1.47%)	2 (1.47%)
*Candida guilliermondii*	1 (1.47%)	1 (1.47%)	2 (1.47%)
*Candida ciferrii*	2 (2.94%)	3 (4.41%)	5 (3.68%)
*Candida lusitaniae*	4 (5.88%)	1 (1.47%)	5 (3.68%)
*Candida parapsilosis*	0 (0%)	11 (16.18%)	11 (8.09%)
**Total**	68 (100%)	68 (100%)	136 (100%)

### Biofilm quantification by crystal violet assay

**Figure 1:**
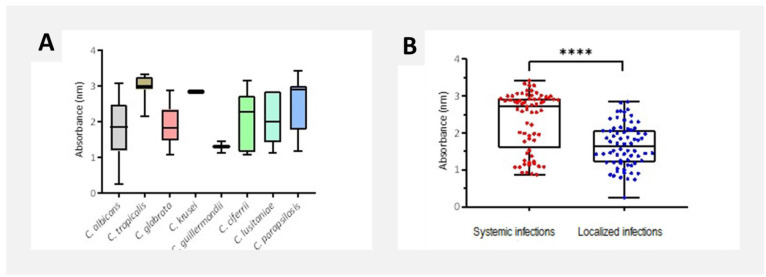
Biofilm quantification by crystal violet assay according to (A) *Candida* species, and (B) infection type. Data are presented as mean ± SD for each isolate in four technical replicates.

*Candida tropicalis* exhibited the highest biofilm mass (2.98 ± 0.33), followed by *C. krusei*, *C. parapsilosis*, and *C. lusitaniae* (Figure 1A). Systemic infection isolates produced significantly more biofilm (2.34 ± 0.79) than localized infection isolates (1.66 ± 0.58; *p* < 0.0001) (Figure 1B).

### Biofilm quantification by XTT reduction assay

**Figure 2:**
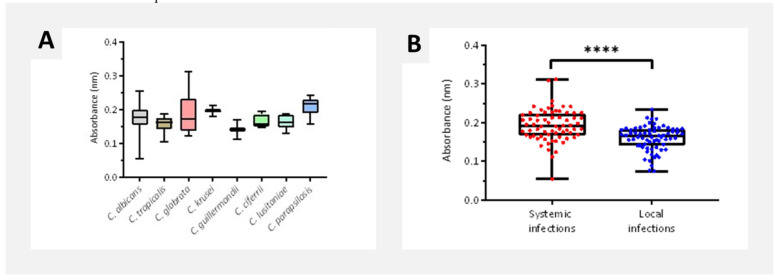
Biofilm metabolic activity quantification by XTT assay according to (A) *Candida* species and (B) infection type. Data are presented as mean ± SD for each isolate in four technical replicates. **p < 0.01 or ***p < 0.001 or ****p < 0-0001.

*Candida parapsilosis* displayed the highest metabolic activity (0.210 ± 0.025), followed by *C. krusei* and *C. glabrata*. Systemic isolates had significantly higher metabolic activity (0.193 ± 0.041) than localized ones (0.160 ± 0.032; *p* < 0.0001) (Figure 2).

### Phospholipase and protease activity evaluation

The mean phospholipase and proteinase production values from the 136 *Candida* isolates by species and infection type are shown in Table 2. Phospholipase activity was ranked based on the following order: *C. guilliermondii* > *C. ciferrii* > *C. glabrata* > *C. albicans* > *C. lusitaniae* > *C. parapsilosis*. Proteinase production showed the trend: *C. guilliermondii* > *C. albicans* > *C. lusitaniae* > *C. ciferrii* > *C. glabrata* > *C. tropicalis* > *C. parapsilosis* > *C. krusei*. Phospholipase activity was detected in 58 (85.29%) localized infection isolates and 30 (44.12%) systemic infection isolates, but no significant difference in phospholipase production was observed between localized (0.622 ± 0.083) and systemic infection isolates (0.663 ± 0.075). Proteinase activity was found in 65 localized and 36 systemic infection isolates, with systemic isolates showing significantly higher activity (0.463 ± 0.182), compared to localized isolates (0.261 ± 0.078) (*p* < 0.001) (Table 3).

**Table 2:** Phospholipase and proteinase activities of Candida clinical isolates according to Candida species. Data are presented as mean ± SD for each isolate in duplicate

Species	Total number	Phospholipase production			Proteinase production		
		Positive	Percentage	*Pz* ± SD	Positive	Percentage	*Pz* ± SD
*C. albicans*	87	78	89.66	0.635 ± 0.084	65	74.71	0.280 ± 0.109
*C. tropicalis*	12	-	-	-	12	100.00	0.536 ± 0.157
*C. glabrata*	12	2	16.67	0.617 ± 0.008	8	66.67	0.307 ± 0.120
*C. krusei*	2	-	-	-	2	100.00	0.600 ± 0.000
*C. guilliermondii*	2	1	50.00	0.558 ± 0.000	1	50.00	0.186 ± 0.000
*C. ciferrii*	5	4	80.00	0.604 ± 0.058	3	60.00	0.298 ± 0.175
*C. lusitaniae*	5	2	40.00	0.700 ± 0.064	5	100.00	0.295 ± 0.154
*C. parapsilosis*	11	1	9.09	0.786 ± 0.000	5	45.45	0.560 ± 0.148

**Table 3:** Phospholipase and proteinase activities of Candida clinical isolates according to infection type. Data are presented as mean ± SD for each isolate in duplicate

**Infection type**	**Isolates assessed**	**Phospholipase production**			**Proteinase production**		
		**Positive**	**Percentage**	***Pz* ± SD**	**Positive**	**Percentage**	***Pz* ± SD**
Local infection	68	58	85.29	0.622 ± 0.083	65	95.59	0.261 ± 0.078
Systemic infections	68	30	44.12	0.663 ± 0.075	36	52.94	0.463 ± 0.182
*p* value				0.0266			< 0.001

### Scanning electron microscopy

**Figure 3:**
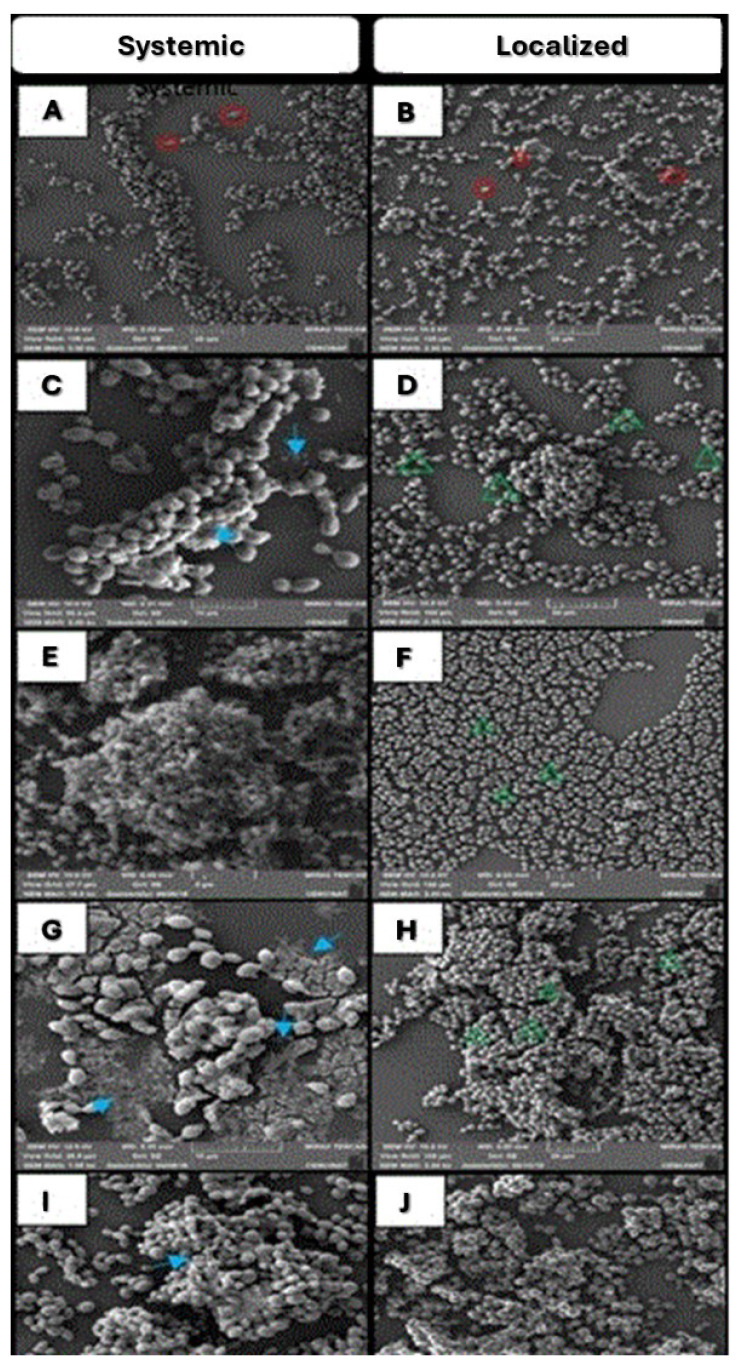
Scanning Electron Microscopy of clinical isolates. Isolates from systemic infections are represented in the left column, and isolates from localized infections are represented in the right column. The isolates tested were (A-B) *C. albicans*, (C-D) *C. ciferrii*, (E-F) *C. glabrata*, (G-H) *C. guilliermondii*, (I-J) *C. lusitaniae*, and (K-L) *C. tropicalis*. Blastoconidial cell poles with budding areas are marked with red circles. The extracellular material is signified with sky blue arrows. Large holes in the middle of aggregated yeast cells are marked with green arrows.

At the biofilm structure level, *C. albicans* biofilms appeared as moderately sized, separated cell aggregates ( Figure 3-A), with more dispersed, non-contiguous clusters throughout the field ( Figure 3-B). *C. ciferrii* biofilms were thicker, with co-aggregated cells and free yeasts scattered across the field ( Figure 3-C, D), featuring extensive extracellular material (ECM) around the main biofilm ( Figure 3-C) and structural holes in some areas ( Figure 3-D). *Candida glabrata* biofilms consisted of large, multilayered cell aggregates covering the surface, with smaller clusters around them ( Figure 3-E). Other isolates formed non-continuous monolayers attached to the surface, with tiny cell aggregates and few individual cells ( Figure 3-F). *Candida guilliermondii* from systemic infections formed poor biofilms with small, separated aggregates and scattered free cells surrounded by ECM ( Figure 3-G). Localized infection isolates formed large, compact multilayer biofilms covering the entire surface, with altered cell morphology near the center ( Figure 3-H). *C. lusitaniae* biofilms were thick with closely packed cells ( Figure 3-I, J). A large biofilm with substantial ECM was visible ( Figure 3-I), while irregularities in cell brightness were observed due to variations in gold coating ( Figure 3-J). *Candida tropicalis* biofilms featured elongated cells forming small clusters across the field ( Figure 3-K, L), with high ECM production around the cells ( Figure 3-K) and large holes within the biofilm structure ( Figure 3-L).

### Confocal laser scanning microscopy

**Figure 4:**
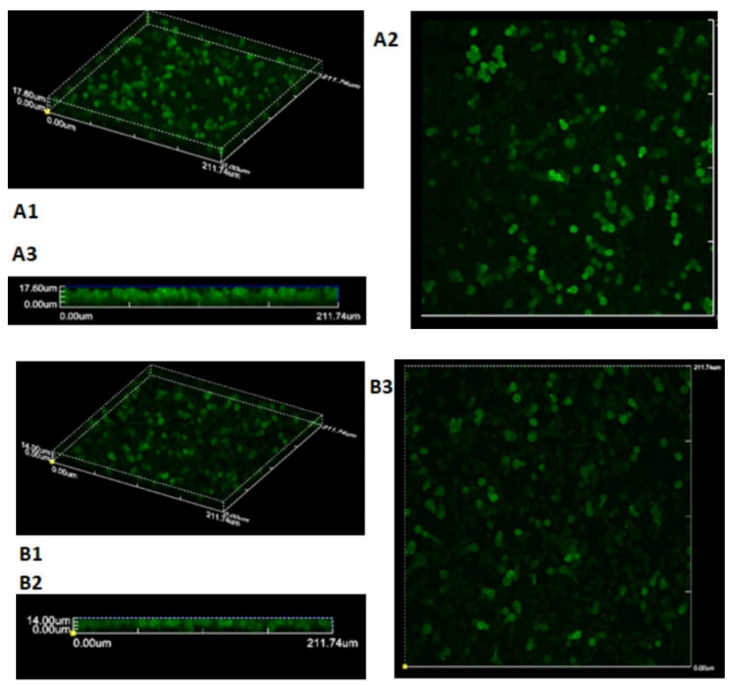
Confocal scanning fluorescence microscopy images of a mature biofilm from a disseminated mycosis *C. albicans* isolate. A1. ConA (10 ug/mL) biofilm fluorescence labelling (60X); A2. Contrast image (60X); A3. Overlapped image and a localized infection isolate. B1. ConA (10 ug/mL) biofilm fluorescence labeling (60X); B2. Contrast image (60X); B3. Overlapped image.

Two *Candida albicans* isolates were selected, one from systemic infections and one from localized infections, to assess differences in biofilm size and structure. Figure 4 presents images of *Candida* cells labeled with Concanavalin A – Alexa Fluor 488 (ConA). The Z-axis scanning revealed biofilm thicknesses of 27.72 and 12.78 µm for the systemic infection isolate and the localized infection isolate, respectively.

## Discussion

In Ecuador, there is not enough data about virulence factors in *Candida* infections. Guerrero *et al*. [ 15 ] identified susceptibility patterns and distribution of *Candida* species isolates from the Instituto Nacional de Investigación en Salud Pública, but studies on fungal infections in Ecuador are still scarce. The frequencies for *Candida* species observed in Table 1 are consistent with the results of other authors [ 3 , 5 , 26 , 27 ]. Tan *et al*. [ 28 ] stated that respiratory infections caused by *C. krusei* are not frequent. There is a higher reported proportion of *C. parapsilosis* in sputum, which is mostly found as a coinfection with tuberculosis [ 26 ]. Pongrácz *et al*. [ 29 ] demonstrated that non-*albicans Candida* species are more frequent in blood samples than in other non-sterile sites, which agrees with the results of the present study (Table 2).

Overall, the bulk biofilm production (Crystal Violet [CV] assay) (Figure 2B) and metabolic activity (XTT assay) (Figure 2B) of disseminated mycosis isolates were notably higher in comparison to those of localized infection isolates (*p* < 0.05), which aligns with the findings of previous studies [ 21 , 30 , 31 ] [ 32 - 34 ]. Meanwhile, most non-*albicans Candida* isolates exhibited significantly higher biofilm formation and metabolic activity, compared to *Candida albicans* isolates (Figures 2-A and 3-A).

Capacity of non-*albicans Candida* isolates to produce more biofilm is important in invasive candidiasis [ 35 ]. Results of the present research corroborate with previous research regarding high bulk biofilm production and low metabolic activity in *C. tropicalis* [ 21 - 33 ]. *Candida parapsilosis* and *C. glabrata* have shown higher metabolic activity than other *Candida* species, with variations in bulk biofilm formation [ 30 , 32 , 36 ]. These findings are in line with those of the present study.

In this study, phospholipase and protease activity were found in one-third of clinical isolates from localized candidiasis. The clinical impact of the enzymatic activity of the non-invasive isolates relies on the strain´s ability to progress toward severe invasive infections, particularly in immunocompromised patients [ 37 ]. Consequently, higher enzyme production indicates higher virulence [ 38 ]. The phospholipase production assay revealed a moderate rate of activity in isolates from systemic infections (44.12%) and a higher rate in isolates from localized infections (85.29%). Similarly, the protease assay yielded fewer isolates showing protease activity of systemic infections (52.94%), compared to those of localized infections (95.59%). Concurrently, the *Pz* value showed that isolates from localized infections have higher production of hydrolytic enzymes than systemic infection isolates (Table 3). Results of the present research concur with those of other studies [ 37 , 39 , 40 ]. Panizo *et. al*. [ 38 , 39 ] found that respiratory tract isolates showed higher rates of enzymatic production, followed by isolates from vaginal secretions and oral cavity. Notably, blood isolates often did not display enzymatic activity. 

However, other authors have found that bloodstream isolates had higher phospholipase and protease activity than isolates of localized infections, with proportions reaching 50% and 75%, respectively [ 38 , 41 ]. Hydrolytic enzyme activity might be limited in blood isolates from suspected catheter- or central intravenous line-associated infection sources [ 42 ]. These isolates might show high biofilm activity, highlighting the role of adhesins in attachment to medical devices. While the role of hydrolytic enzymes in tissue penetration is well known, their activity may be influenced by the infection source and the immune status of the patient [ 43 ]. However, some studies have reported strain-specific protease and phospholipase activity, and overall virulence patterns [ 44 ].

On SEM visualization, mature biofilms of *C. albicans* showed complex, confluent blastoconidial layers covered with ECM, with the absence of hyphal elements [ 45 , 46 ]. Previous reports with XTT and CV assays [ 47 ] have confirmed the low biofilm production characteristic of *C. guilliermondii* ( Figure 3-G). Paiva *et al*. [ 48 ] have reported the typical adherence to the surface of blastoconidia. On the other hand, and in agreement with other authors [ 46 , 49 ], it was found that *C. lusitaniae* biofilms exhibit thick layers, without filaments but with high adherence. According to Silva *et al*. [ 33 ], Thein *et al*. [ 50 ], and Camarillo *et al*. [ 51 ], *C. glabrata* shows a multilayered structure with yeast cells intimately packed and typically without hyphae and pseudohyphae (*C. glabrata* is a non-hyphal species). These findings are consistent with those of the present study (Figure 4E). *Candida tropicalis* isolates developed co-aggregated microcolonies of yeast cells with a prominent ECM layer ( Figure 3K and 4L). Results of the present research corroborate with those of a study performed by Parahitiyawa *et al*. [ 52 ] where *C. tropicalis* presented the same structure on polystyrene surfaces.

Confocal scanning microscopy studies have shown that *C. albicans* produces larger biofilms in comparison to other pathogenic species. Some biofilms have been measured up to 450 µm thick in 24-72 h visualizations [ 19 , 20 , 45 , 53 , 54 ]. Though only two isolates that were cultured under similar conditions were analyzed in this study, Z-size measurements were different, with the biofilm formed from the invasive isolate being thicker than the one from the localized infection isolates. Kuhn *et al*. [ 53 ] found that *Candida albicans* isolates in general form more biofilm when comparing the dry weight of the biofilm from invasive and non-invasive isolates.

## Conclusion

The present study is the first, to our knowledge, to investigate the virulence factors of pathogenic *Candida* species in Ecuador. Differences in enzymatic activity may depend on the site and source of infection, the immune status of the patient, and strain-specific variation. This highlights the importance of considering the infection context when assessing virulence. The SEM analysis and confocal microscopy confirmed differences in biofilm size and structure between isolates from systemic and localized infections. Overall, this research contributed to the limited scientific literature on Candida infections in Ecuador and emphasized the need for ongoing research and surveillance in regions with scarce data. 
